# Potential Mechanisms Underlying Inflammation-Enhanced Aminoglycoside-Induced Cochleotoxicity

**DOI:** 10.3389/fncel.2017.00362

**Published:** 2017-11-21

**Authors:** Meiyan Jiang, Farshid Taghizadeh, Peter S. Steyger

**Affiliations:** ^1^Oregon Hearing Research Center, Oregon Health & Science University, Portland, OR, United States; ^2^National Center for Rehabilitative Auditory Research, VA Portland Health Care System, Portland, OR, United States

**Keywords:** aminoglycosides, gentamicin, ototoxicity, sepsis, infection, bacteriogenic, virogenic, inflammation

## Abstract

Aminoglycoside antibiotics remain widely used for urgent clinical treatment of life-threatening infections, despite the well-recognized risk of permanent hearing loss, i.e., cochleotoxicity. Recent studies show that aminoglycoside-induced cochleotoxicity is exacerbated by bacteriogenic-induced inflammation. This implies that those with severe bacterial infections (that induce systemic inflammation), and are treated with bactericidal aminoglycosides are at greater risk of drug-induced hearing loss than previously recognized. Incorporating this novel comorbid factor into cochleotoxicity risk prediction models will better predict which individuals are more predisposed to drug-induced hearing loss. Here, we review the cellular and/or signaling mechanisms by which host-mediated inflammatory responses to infection could enhance the trafficking of systemically administered aminoglycosides into the cochlea to enhance the degree of cochleotoxicity over that in healthy preclinical models. Once verified, these mechanisms will be potential targets for novel pharmacotherapeutics that reduce the risk of drug-induced hearing loss (and acute kidney damage) without compromising the life-saving bactericidal efficacy of aminoglycosides.

## Introduction

In the United States, 12% (∼480,000) of ∼4 million live births are admitted into the neonatal intensive care unit (NICU) each year (Osterman et al., 2011). NICU patients with confirmed sepsis, or those who develop necrotizing enterocolitis, receive aminoglycosides, typically gentamicin, for 7–10 days or more (Remington, 2011; Blackwood et al., 2017). Yet, clinical use of aminoglycosides carries the risk of permanent hearing loss (cochleotoxicity) that is dose-dependent in preclinical models, and/or acute kidney injury (Forge and Schacht, 2000). The incidence of hearing loss in infants discharged from the NICU ranges between 2 and 15%, compared to 0.3% for full-term babies (Yoon et al., 2003). One factor for this differential prevalence could be cumulative dosing with intravenous administration of aminoglycosides (Garinis et al., 2017c). Another aminoglycoside, tobramycin, induces dose-dependent hearing loss in older pediatric and adult patients with cystic fibrosis that experience repeated severe respiratory infections (Al-Malky et al., 2015; Garinis et al., 2017a). The majority of adults with multi-drug resistant tuberculosis chronically treated with aminoglycosides, typically amikacin or kanamycin over many months, experience permanent hearing loss in a dose-frequency dependent manner (Sagwa et al., 2015).

Only recently have preclinical ototoxicity studies incorporated experimentally induced inflammation (mimicking clinical infections), and found enhanced cochleotoxicity over that in untreated, healthy animals (Oh et al., 2011; Hirose et al., 2014b; Koo et al., 2015). Yet, bacteriogenic induction of experimental systemic sepsis (excluding meningitis and labyrinthitis) has little direct impact on auditory function (Hirose et al., 2014b; Koo et al., 2015). This strongly indicates that systemic inflammatory responses represent a novel co-morbidity that enhances ototoxicity, alongside other better characterized factors such as age, mitochondrial polymorphisms, acoustic trauma, renal dysfunction, and co-therapeutics like loop diuretics or vancomycin (Forge and Schacht, 2000; Garinis et al., 2017b; Jiang et al., 2017). Identifying the factors associated with infection-induced inflammation that increase the risk of aminoglycoside-induced hearing loss will promote new clinical strategies to ameliorate drug-induced ototoxicity. Here, we postulate several mechanisms by which systemic inflammation could exacerbate aminoglycoside-induced cochleotoxicity.

## Systemic Inflammation Enhances Aminoglycoside-Induced Cochleotoxicity

**FIGURE 1:**
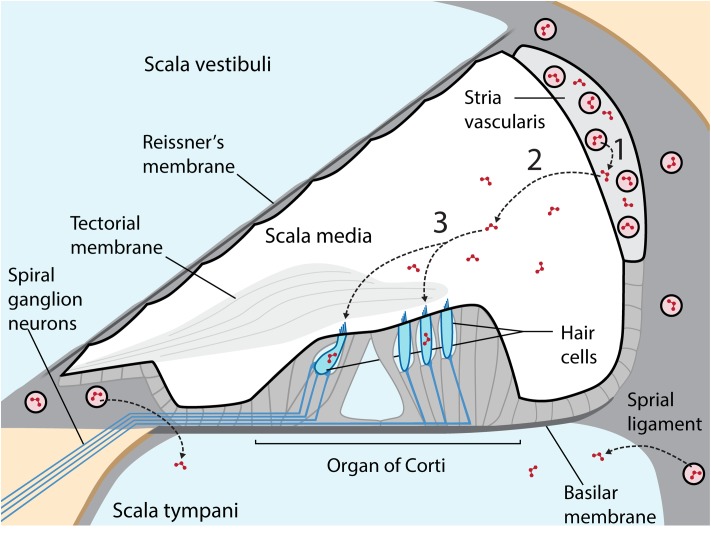
Cross-section of the cochlear duct, with perilymph-filled scala vestibuli and scala tympani (pale blue) separated from the endolymphatic scala media (white) by tight junctions between adjacent cells (thicker black line) in Reissner’s membrane, the stria vascularis (light gray) and reticular lamina of the organ of Corti on the basilar membrane. Within the organ of Corti are four longitudinal rows of sensory hair cells (blue), under the tectorial membrane, and innervated by afferent and efferent fibers (blue lines). The highly vascularized stria vascularis has capillaries (pink circles) lined by tight junction-coupled endothelial cells (black lines enclosing pink circles) that form the BLB. Circulating aminoglycosides preferentially cross the BLB into the stria vascularis (1) and are cleared into endolymph (2) prior to entering hair cells across their apical membranes (3). Aminoglycosides also enter perilymph, but this trafficking route is not a major contributor to hair cell uptake in healthy guinea pigs. Diagram not to relative scale, and adapted with permission from Macmillan Publishers Ltd., Li and Steyger (2011).

Aminoglycosides are primarily administered systemically to resolve life-threatening bacterial infections that trigger systemic, host-mediated inflammatory responses that rapidly lead to mortality without medical intervention (Mahmoudi et al., 2013). Circulating aminoglycosides readily cross the cochlear blood-labyrinth barrier (BLB) to preferentially load the highly vascularized stria vascularis, and are cleared into endolymph (**Figure 1**). The apical membranes of cochlear hair cells are immersed in endolymph with an electrical potential of +80 mV, while the resting potential of inner and outer hair cells are –45 and –70 mV, respectively (Pickles, 2012). This high potential difference (∼135–150 mV) produces a significant electro-repulsive force to drive the cationic aminoglycosides, from endolymph across the apical membranes of hair cells into their electrically negative cytoplasm (Marcotti et al., 2005; Li and Steyger, 2011), with consequent cytotoxic effects (Hiel et al., 1993).

**FIGURE 2:**
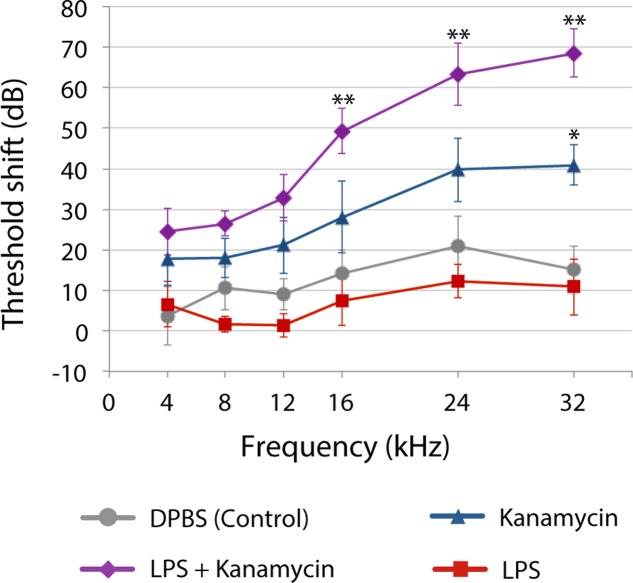
Three weeks after chronic [lipopolysaccharides (LPS) or saline] exposure with or without twice daily kanamycin dosing, ABR threshold shifts for mice treated with LPS-only (red) were not different from saline-treated mice (DPBS, gray). Kanamycin alone (700 mg/kg, twice daily; blue) induced a small but significant PTS at only 32 kHz (^∗^*P* < 0.01) compared to saline-treated mice (gray). that received LPS plus kanamycin (purple) had significant PTS at 16, 24 (^∗∗^*P* < 0.01), and 32 kHz (*P* < 0.05) compared to mice treated with kanamycin, saline or LPS only (^∗∗^*P* < 0.01). Mice receiving LPS plus kanamycin also had significant PTS at 12 kHz compared to mice treated with DPBS or LPS only, or LPS-only mice at 8 kHz. Error bars = SD. Figure adapted from Koo et al., 2015, with permission from Science/American Association for the Advancement of Science.

Yet, until recently, most preclinical studies of aminoglycoside-induced cochleotoxicity used healthy preclinical models (Wu et al., 2001; Roy et al., 2013; Duscha et al., 2014). Systemic models of inflammation that mimic infection mediate physiological changes in the blood-brain barrier permeability (Abbott et al., 2006). Bacteriogenic induction of systemic inflammation during chronic aminoglycoside dosing increased the range of frequencies with significant permanent auditory threshold shifts (PTS; **Figure 2**), and extent of outer hair cell death compared to age-matched mice treated with kanamycin alone or saline (Koo et al., 2015). Bacteriogenic induction of systemic inflammation also exacerbated both combinatorial kanamycin/loop diuretic-induced, and also cisplatin-induced, cochleotoxicity (Oh et al., 2011; Hirose et al., 2014b).

A pilot study of NICU subjects (91 subjects) revealed that those with (suspected) sepsis and gentamicin therapy for ≥5 days (18 subjects; 20%) were twice as likely to be referred on a distortion product otoacoustic emission hearing screen compared to all other subjects (Cross et al., 2015). Cystic fibrosis patients with lower lung function scores (indicative of respiratory infection and inflammation) were also more likely to experience cochleotoxicity (Pillarisetti et al., 2011; Al-Malky et al., 2015). The mitochondrial polymorphism most associated with aminoglycoside-induced hearing loss (mt1555A > G) has an incidence between 0.09 and 0.2% (Tang et al., 2002; Bitner-Glindzicz et al., 2009), two orders of magnitude less, and unlikely to statistically influence the number of referred neonates in these studies. Thus, there is an increased risk of drug-induced hearing loss in those receiving aminoglycoside therapy for bacterial infections. Furthermore, 20% of live births with confirmed infection are viral in etiology, yet these infants are empirically treated with aminoglycosides until the causative agent is identified (Remington, 2011). It will be important to determine whether virogenic-induced inflammation enhances cochlear uptake of aminoglycosides and exacerbates cochleotoxicity. To better understand how inflammation could increase cochlear uptake of aminoglycosides, we need to explore inflammatory signaling prior to discussing their potential effect on cochlear uptake mechanisms.

### Bacteriogenic and Virogenic Inflammatory Signaling Cascades

**FIGURE 3:**
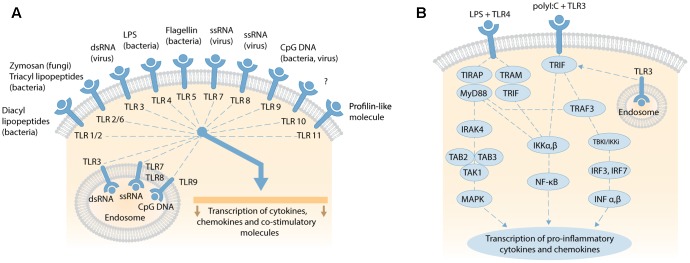
**(A)** Overview of human TLRs activated by exogenous and endogenous ligands, such as fragmented DNA from necrotic cells; adapted by permission from Macmillan Publishers Ltd, Nature Reviews Gastroenterology and Hepatology, 2006, vol. 3, pp..390–407, Sartor (2006). **(B)** Schematic of TLR4 and TLR3 signaling pathways. LPS binding to membranous TLR4 activates the MyD88-dependent and MyD88-independent signaling pathways via different adaptor proteins. MyD88-dependent pathway activates IRAK-4, transforming TAK1 and TAK-binding protein 2 or 3 (TAB2/3) to stimulate downstream MAPK, and transcription and expression of pro-inflammatory cytokines (e.g., TNFα, IL-1α, IL-1β, IL-2, IL-6, IL-12). The MyD88-independent pathway activates IκB kinase (IKK) complex, releasing NF-κB to translocate to the nucleus and transcribe genes that express type 1 interferons. Viral double-stranded (dsRNA) binds TLR3 on cell or endosomal membranes and recruit the adaptor molecule TRIF. This initiates two pathways via IKKα,β and TRAF-3. IKKα,β activates NF-κB subunits which translocate to the nucleus to trigger transcription of genes encoding pro-inflammatory cytokines. Alternatively, TRIF stimulates TRAF3 to activate TBK1/IKKi and phosphorylate transcription factor IRF-3 and IRF-7. After homodimerization, IRF-3 and-IRF-7 translocate to the nucleus to transcribe type I IFNα,β. Secretion of type 1 IFNα,β leads to further transcription and expression of pro-inflammatory cytokines. Both schematics are not to scale.

Bacterial and viral penetration of blood, tissues and interstitial fluids are typically detected by Toll-like receptors (TLRs) that trigger inflammatory signaling cascades to induce an overwhelming immune response to reduce the risk of pathogenic infection. TLRs are highly conserved pattern-recognition receptors present in diverse cell types, including immune, endothelial, epithelial, and fibrocytes (Atkinson, 2008). There are currently 11 human (and 13 mammalian) TLRs that share common transmembrane domains with leucine-rich repeats that bind to an overlapping array of extracellular (or endosomal) ligands, and a cytosolic signaling domain – the Toll-IL-1 Receptor (TIR) domain (**Figure 3A**). Here, we briefly review the signaling cascades activated by TLR4, the most studied TLR, and also TLR3.

TLR4 (a.k.a CD284, cluster of differentiation 284), was the first to have its specific ligand defined–lipopolysaccharides (LPS) from the cell wall of Gram-negative bacteria (Poltorak et al., 1998). TLR4 is constitutively expressed on the plasma membranes of monocytes, T cells, B cells, and dendritic cells, with induced expression in non-hematopoietic cells (Chakravarty and Herkenham, 2005). Extracellular, soluble LPS-binding protein (LBP) extracts LPS monomers from aggregates released from lyzed bacteria (Schumann et al., 1990). Bound LPS then complexes with CD14 (cluster of differentiation 14), a membrane-anchored glycoprotein, and extracellular lymphocyte antigen 96 (also known as MD2) to activate TLR4 (Shimazu et al., 1999). The complex facilitates picomolar detection of LPS, otherwise millimolar levels of LPS are required to activate TLR4 directly.

Activated TLR4 triggers one or more TIR domain-containing signaling adaptors: Myeloid Differentiation Primary Response Gene 88 (MyD88), TIR Domain-Containing Adaptor Protein (TIRAP), TIR-domain-containing adaptor inducing interferon-β (TRIF), and TRIF-related Adaptor Molecule (TRAM) that activate individual signaling cascades (Kawai and Akira, 2010; Kim and Sears, 2010; Juskewitch et al., 2012; Hamerman et al., 2016). These cascades are divided into MyD88-*dependent* (MyD88 and TIRAP), and MyD88-*independent* (TRIF and TRAM) signaling cascades (**Figure 3B**). The MyD88-*dependent* pathway signals through IL-1 receptor-associated kinase (IRAK)-4, transforming growth factor-β-activated kinase (TAK) 1, and TAK-binding protein 2 or 3 (TAB2/3) to activate mitogen-activated protein kinase (MAPK) downstream. This in turn leads to the transcription and expression of pro-inflammatory cytokines, such as TNFα, IL-1α, IL-1β, IL-6, or alternatively apoptosis (Takeuchi and Akira, 2009; Guo and Friedman, 2010). The MyD88-*independent* pathway activates IκB kinase (IKK) complex, releasing NF-κB for translocation to the nucleus and transcription of genes for expression of type 1 interferons (O’Neill et al., 2013). Another, complex TIR-domain-containing adaptor protein called Sterile α and HEAT (Armadillo motif; SARM) inhibits the TRIF-mediated (MyD88-*independent*) pathway, and attenuates LPS-mediated signaling to dampen inflammation and abrogate septic shock and multiple organ dysfunction syndrome (Aird, 2003). The time-course of plasma/serum expression profiles for individual acute phase inflammatory (API) cytokines and chemokines changes over hours and days following induction of the inflammatory response (Allan and Rothwell, 2001; Juskewitch et al., 2012).

Viral double-stranded DNA (dsDNA), mRNA, ionizing radiation or hypoxia can activate the ubiquitously expressed TLR3, located on both cell and endosomal membranes, to activate an adaptor protein called TRIF (**Figure 3B**) (Zarember and Godowski, 2002; Kawai and Akira, 2010). TRIF initiates two pathways via IKKα,β and TRAF-3. IKKα,β activates NF-κB subunits that translocate to the nucleus to initiate the transcription of genes for API cytokines and chemokines induced by MyD88 signaling, with differing expression profiles over time (Alexopoulou et al., 2001; Lien and Zipris, 2009; Kawai and Akira, 2010; Kishimoto, 2010). TRAF3 activates TBK1/IKKi to phosphorylate and homodimerize the transcription factors IRF-3 and IRF-7. These dimers then translocate to the nucleus to induce the expression of type I interferon-α (IFN-α [13 subtypes]) and IFN-β. After secretion, these interferons induce the expression of pro-inflammatory cytokines (Assmann et al., 2015). The IFN-α family, and IFN-β, influence a vast spectrum of biological functions, including inhibition of viral replication (Borden et al., 2007), and regulating the homeostatic differentiation of natural killer cells, dendritic cells, B cells, T cells, and osteoclasts (Farrar and Murphy, 2000). Activated IFN-β also phosphorylates the signal transducers and activators of transcription 1 (STAT1) protein (Imaizumi et al., 2016a). IFN-stimulated genes (ISGs) then induce and modulate various biological processes, especially anti-viral activities that target almost all steps in the lifecycle of a virus (Imaizumi et al., 2016a,b).

## Experimental Models of Systemic Infection and Cochlear-Mediated Inflammatory Responses

Experimental models of infection allow researchers to identify the effect of induced inflammation on normal physiology, a rapidly growing area of research. Classic experimental models of infection use parenteral administration of LPS or polyinosinic:polycytidylic acid (polyI:C) to induce innate immune responses. LPS (a.k.a lipoglycans or endotoxin) is a potent bacteriogenic agonist for TLR4 (Nemzek et al., 2008). PolyI:C is synthetic dsRNA that primarily binds to TLR3, stimulating an innate virogenic immune response (Fortier et al., 2004). The experimental advantages of using LPS and polyI:C as immunogenic stimulants include safety, convenience, control over dose and administration of the immunological challenge, and more importantly reproducibility between individuals within the same group compared to that achieved by inoculation with live bacteria and viruses. The complex interplay between live bacteria or viruses and host immune responses to can lead to wide-ranging experimental outcomes within the same group. LPS-induced inflammation is characterized by time-dependent levels of individual cytokines that are less sustained compared to live bacterial models with polymodal avenues of immunostimulation (Hadjiminas et al., 1994; Nemzek et al., 2008).

The innate immune (inflammatory) response includes secretion of nitric oxide and bacteriotoxic enzymes by immune cells (monocytes, macrophages, neutrophils etc.) that lyze bacteria. Aminoglycosides also lyze bacteria (Martin and Beveridge, 1986; Kadurugamuwa et al., 1993). Lysis of Gram-negative bacteria releases LPS that further stimulates the TLR4-mediated immune response, heightening the systemic host-mediated inflammatory response, analogous to the Jarisch–Herxheimer reaction following penicillin treatment for syphilis (Shenep and Mogan, 1984; Kaplanski et al., 1998; Yang et al., 2010).

Initially, the inner ear was considered an immuno-privileged organ that did not participate in the systemic inflammatory responses (Fujioka et al., 2014). Of 458 articles on cochlear inflammation indexed by PubMed, more than 55% were published in the last 10 years (search conducted June 2017). It is now widely recognized that cochlear inflammation can recruit immune cells into the cochlea (Hirose et al., 2005; Miyao et al., 2008) and, also repair and resolve cochlear damage, as described elsewhere in this Research Topic (Kalinec et al., 2017; Wood and Zuo, 2017).

Experimental models of systemic inflammation were only recently incorporated into preclinical ototoxicity studies (Koo et al., 2011; Quintanilla-Dieck et al., 2013). Crucially, systemic LPS does not significantly modulate the cochlear endolymphatic potential or auditory function (Hirose et al., 2014b; Koo et al., 2015), yet altered BLB physiology that facilitated increased entry of fluorescent markers into perilymph by mechanisms that remain to be directly identified (Hirose et al., 2014a). Systemic administration of also LPS increases cochlear levels of aminoglycosides, particularly in the stria vascularis, without modulating serum levels for these drugs. Furthermore, systemic LPS increased the expression of acute phase inflammatory markers in both serum, and, surprisingly, in cochlear tissues that was not replicated in mice with hypofunctional TLR4 (Koo et al., 2015).

PolyI:C significantly enhances the secretion of thymic stromal lymphopoietin (TSLP), B lymphocyte stimulator (BLyS), IFNγ-inducible protein 10 (IP-10), and macrophage inflammatory protein 1 alpha (MIP-1α) in human inner ear endolymphatic sac fibroblasts (Yamada et al., 2017). This suggests that cells in the endolymphatic sac can also produce cytokines and chemokines in response to activated TLR3 (Yamada et al., 2017). Inoculation of cochleae with live or heat-inactivated *Cytomegalovirus* altered BLB permeability, and induced recruitment of inflammatory cells to the spiral ligament, with cochlear inflammation and degeneration present after 5 weeks (Keithley et al., 1989; Fukuda et al., 1992; Keithley and Harris, 1996; Li et al., 2014).

## Potential Mechanisms Underlying Inflammation-Enhanced Cochleotoxicity

In the stria vascularis, peri-vascular resident macrophages are thought to modulate the integrity of the strial BLB (and inversely, paracellular flux). The loss of these macrophages decreased the endolymphatic potential, elevated auditory thresholds and increased paracellular flux into the stria vascularis (Zhang et al., 2012). In other tissues, systemic inflammation is associated with decreased expression of tight junctional proteins and increased permeability (Hofer et al., 2008; Singla et al., 2011; Yun et al., 2017). Preclinical models of a disrupted BLB (loss of physical integrity) also results in loss of the endolymphatic potential, elevated auditory thresholds and increased protein expression of genes for ion homeostasis and junctional complexes (Lin and Trune, 1997; Trune, 1997; MacArthur et al., 2006; Cohen-Salmon et al., 2007; MacArthur et al., 2013). However, there is no loss of endolymphatic potential, nor elevated auditory thresholds, during systemic inflammation induced by (lower doses of) LPS that enhanced cochleotoxicity, suggesting that the BLB remained relatively intact (Hirose et al., 2014b; Koo et al., 2015).

For systemically administered aminoglycosides to reach cochlear hair cells, these drugs must first enter endothelial cells forming the BLB, established by tight junctions between adjacent endothelial cells of cochlear blood vessels. The most intense strial uptake of fluorescent gentamicin is within endothelial cells of the strial capillaries, and this uptake can be attenuated by increasing levels of unconjugated aminoglycosides, suggestive of competitive antagonism of saturable cell-regulatable mechanisms (Wang et al., 2010). Aminoglycosides can use one or more cell-regulatable transcellular trafficking routes, including endocytosis and/or permeation through ion channels (e.g., TRPV4) to enter cochlear endothelial cells that form the BLB (Koo et al., 2015).

**FIGURE 4:**
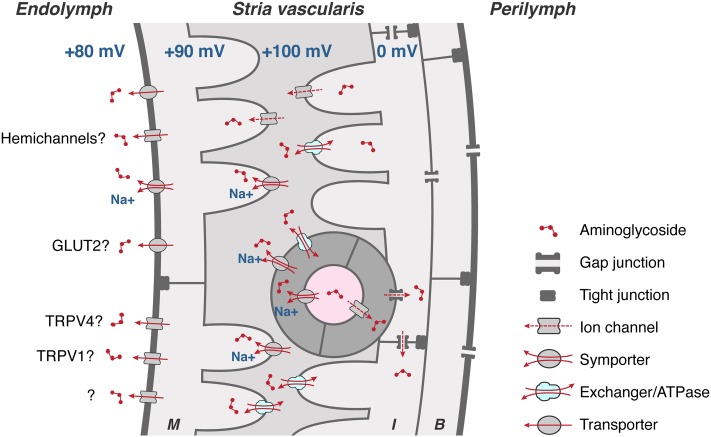
To cross the strial BLB, aminoglycosides must first enter endothelial cells (dark gray), and permeate through gap junctions into intermediate cells (I) and/or basal cells (B). Aminoglycosides could clear endothelial, intermediate and basal cells via transporters, exchangers, and/or cation channels, or by exocytosis of endosomes (not shown), into the intra-strial space. Aminoglycosides are taken up by marginal cells across their basolateral membranes, presumptively by ATPases, exchangers, and transporters (and ion channels?). Once in marginal cells, aminoglycosides clear into endolymph down the electrochemical gradient, presumptively via permeation of hemi-channels, facilitated glucose transporters (GLUT), electrogenic symporters, and at least two TRP channels, TRPV1 and TRPV4. Schematic diagram not to relative scale.

Aminoglycosides must also be able to exit BLB endothelial cells, and then traffic through the tight junction-coupled marginal cells of the stria vascularis into endolymph (**Figure 4**) prior to entering hair cells across their apical membranes via the aminoglycoside-permeant mechanoelectrical transduction channel. Current flow through most ion channels is passively bi-directional, dependent on the electrophysiological characteristics in which they are situated, e.g., K_ir_4.1 in strial intermediate cells (Ando and Takeuchi, 1999; Marcus et al., 2002), which could facilitate trafficking of aminoglycosides in a similar manner in or out of individual cells within the stria vascularis. Below, we discuss several transmembrane mechanisms that could physiologically modulate the trafficking of the cationic, hydrophilic aminoglycosides.

### Endocytosis

Aminoglycosides are readily endocytosed by specific and non-specific mechanisms (Myrdal et al., 2005). Megalin and cubulin are apical membrane receptors that can bind to aminoglycosides to induce endocytosis and are expressed in renal and cochlear epithelia, but not in hair cells (Tauris et al., 2009; Nagai and Takano, 2014). Mice lacking megalin show reduced renal uptake of aminoglycosides and attenuated aminoglycoside-induced nephrotoxicity (Nagai et al., 2001; Schmitz et al., 2002), and may represent a partial otoprotective mechanism by sequestering aminoglycosides from endolymph (Tauris et al., 2009). However, blocking endocytosis did not reduce hair cell death *in vitro* (Alharazneh et al., 2011). Blocking trafficking of aminoglycoside-laden endosomes to lysosomes exacerbates hair cell death suggesting that aminoglycoside-induced cytotoxicity proceeds upstream of endosomal and lysosomal activity, which may be partially cytoprotective (Esterberg et al., 2014; Hailey et al., 2017). Although inflammation enhances cochlear uptake of aminoglycosides across the BLB of cochlear endothelial cells, it remains to be determined if inflammation modulates transcytosis of aminoglycosides, especially when LPS exposure can reduce caveolin-mediated endocytosis in lung endothelial cells (Singla et al., 2011).

Endothelial cells and macrophages readily endocytose pathogens and particulates which induce inflammatory responses that further induce endocytotic processes (Majkova et al., 2010; Utech et al., 2010). Binding of the LPS-binding proteins complex to TLR4 induces endocytosis and induction of cytokine expression, as described above. Furthermore, this ligand-receptor binding is also endocytosed with downstream production of cytokines (Tan et al., 2015). In preclinical models, treatment with antibodies to TLR2 and TLR4 attenuate the inflammatory response and promote survival of severe experimental sepsis; however, side-effects include delayed healing from infection (Lima et al., 2015; Gao et al., 2017). Etanercept, an antibody that attenuates the TNFα-mediated inflammation triggered by TLR4, can acutely maintain cochlear blood flow and preserve hearing following acoustic overstimulation that typically induces cochlear inflammation (Arpornchayanon et al., 2013), and also cisplatin-induced cochleotoxicity (Kaur et al., 2011). Investigation of off-target side-effects will be crucial to determine the efficacy and safety of these approaches.

### Ion Channels

Any non-selective cation channel on the apical plasma membrane of hair cells (and supporting cells) bathed by endolymph, with a pore diameter larger than the maximum cross-sectional diameter of aminoglycosides (∼0.8–0.9 nm), is a candidate ion channel permeant to these drugs. These include the mechanoelectrical transducer (MET) channel of hair cells, and a variety of Transient Receptor Potential (TRP) channels, expressed by hair cells and supporting cells. There are seven subfamilies of TRP channels (TRPC, TRPM, TRPV, TRPA, TRPP, TRPML, and TRPN; all of which are found in mammals except for TRPN). At least four subfamilies are expressed in the cochlea, of which three subfamilies (TRPA, TRPC, TRPV) have a pore diameter larger than the maximum cross-sectional diameter of aminoglycosides, but not the fourth subfamily (TRPML). The very low concentration of calcium ions in endolymph increases the open probability of these non-selective cation channels, enhancing their permeability to aminoglycosides (Marcotti et al., 2005; Myrdal and Steyger, 2005; Karasawa et al., 2008; Banke, 2011). Furthermore, TRP channels can mediate inflammatory responses through multiple mechanisms, including interactions with other TRP channels, immunological receptors (e.g., TLR4) and signaling molecules such as pro-inflammatory cytokines (Numata et al., 2011). These are discussed below.

### MET Channels

MET channels are big, multi-subunit complexes, including TMC1 and TMC2 (Kawashima et al., 2011), whose interactions are currently being unraveled, and subject to much debate. Nonetheless, their electrophysiological properties are well-characterized and many accessory components identified (Farris et al., 2006). The MET channels are permeable to a variety of aminoglycosides, including fluorescently tagged aminoglycosides (Marcotti et al., 2005; Coffin et al., 2009; Alharazneh et al., 2011; Vu et al., 2013). Genetic disruptions of essential components of the MET complex, e.g., myosin VIIa, or cadherin-23, reduce aminoglycoside uptake (Richardson et al., 1997; Vu et al., 2013). The conductance of MET channels, and therefore aminoglycoside permeation, can readily be modulated by extracellular cations, and permeant or impermeant MET channel blockers, e.g., tubocurarine, quinine (Farris et al., 2004; Coffin et al., 2009; Alharazneh et al., 2011), and are discussed elsewhere in this Research Topic (Kirkwood et al., 2017; O’Sullivan et al., 2017). The intracellular modulation of the MET channel current by inflammatory signaling (or by any other factors) remains to be determined and, if present, will have wider functional implications besides drug permeation into hair cells.

### TRPA1

Transient Receptor Potential Ankyrin 1 (TRPA1) is an inflammatory, irritant, and oxidative stress sensor and has been indirectly localized to the basolateral membrane of outer hair cells (Kwan et al., 2006; Stepanyan et al., 2011). TRPA1 has a pore diameter of 1.1 nm, is dilatable to ∼1.4 nm, and is permeable to organic cations under the effect of agonists, see **Tables 1** and **2** (Chen et al., 2009; Karashima et al., 2010; Banke, 2011). TRPA1 channels are required for the release of inflammatory neuropeptides and are activated by inflammatory agents released by damaged or diseased non-neuronal cells (Bautista et al., 2013). TRPA1 channels can be sensitized by inflammatory signals such as protein kinase A (PKA) and phospholipase C (PLC), which can include translocation of TRPA1 from vesicular stores to the plasma membrane (Schmidt et al., 2009). Endogenous TRPA1 agonists, such as methylglyoxal, 4-hydroxynonenal (4-HNE, a product and inducer of oxidative stress), 12-lipoxygenase-derived hepoxilin A3, 5,6-epoxyeicosatrienoic acid and reactive oxygen species (**Table 2**), are generated under various pathophysiological conditions activate TRPA1, contributing to peripheral neurogenic inflammation (Koivisto et al., 2014). *In vitro* experiments show that TRPA1 agonists, cinnamaldehyde, and 4-HNE increase outer hair cell uptake of fluorescent gentamicin (Myrdal and Steyger, 2005; Stepanyan et al., 2011). Thus, insults that induce oxidative stress in outer hair cells could potentially activate basolateral TRPA1 channels to enhance aminoglycoside uptake from the perilymphatic scala tympani, another depository of aminoglycosides *in vivo* (Tran Ba Huy et al., 1986; Ohlemiller et al., 1999). A cochlear expression map for TRPA1 is required to determine its potential involvement in inflammation enhanced cochlear uptake of aminoglycosides.

**Table 1:** Regulators of TRP channels, including chemical agents, cytokines, and chemokines.

	Thermosensitivity	Translocation	Mechanostimulation	Citations
TRPA1	<17°C	Yes	No	Bandell et al., 2004; Rugiero and Wood, 2009; Takahashi and Ohta, 2017
TRPV1	>43°C	Yes	Yes (splice variant)	Caterina et al., 1997; Ji et al., 2002; Sharif Naeini et al., 2006
TRPV4	33°C; >45°C	Yes	Yes	Cohen, 2005; Hartmannsgruber et al., 2007; Sokabe and Tominaga, 2010; Ma et al., 2011
TRPC3/6	None	Yes	Yes	Mio et al., 2007; Goel and Schilling, 2010; Quick et al., 2012; Hanson et al., 2015


**Table 2:** Regulators of TRP channels, including chemical agents, cytokines, and chemokines.

	Agonists	Antagonists	Citations
TRPA1	Nicotine; mustard oil; cinnamaldehyde (bimodal); cannabinoids; tear gases; zinc; ginger; garlic; 4-HNE; allyl isothiocyanate (AITC); methylglyoxal; 12-lipoxygenase-derived hepoxilin A3; 5,6-epoxyeicosatrienoic acid; reactive oxygen species; bradykinin	HC-030031; AP18; camphor (bimodal)	Bandell et al., 2004; Jordt et al., 2004; McMahon and Wood, 2006; Hu et al., 2009; Talavera et al., 2009; Cao et al., 2012; Sisignano et al., 2012; Alpizar et al., 2013; Koivisto et al., 2014
TRPV1	Anandamide; NADA; capsaicin; piperin; protons; nerve growth factor (sensitization); IL-1β; IL-6; TNFα	Capsazepine; AMG 9810; AMG 517; 5’-iodo-resiniferatoxin	Ahern, 2003; Bonnington and McNaughton, 2003; Reilly et al., 2003; Petho et al., 2004; Gavva et al., 2005; McNamara et al., 2005; Doherty et al., 2007; Schafers and Sorkin, 2008; Hsu et al., 2009; Miller et al., 2009; Lawton et al., 2017
TRPV4	4α-Phorbol 12,13-didecanoate; epoxyeicosatrienoicacids; bisandrographolide; GSK1016790A	Ruthenium Red; Gd^3+^; La^3+^; RN-1734	Vriens et al., 2004; Becker et al., 2005; Smith et al., 2006; Thorneloe et al., 2008; Mendoza et al., 2010; Mergler et al., 2011; Zheng et al., 2013
TRPC3/6	Diacylglycerol; GSK1702934A	GSK417651A; GSK2293017A	Hofmann et al., 1999; Mio et al., 2007; Goel and Schilling, 2010; Quick et al., 2012; Tauseef et al., 2012; Chaudhuri et al., 2016


### TRPV1

The Transient Receptor Potential Vanilloid (TRPV) subfamily includes TRPV1, the first TRP channel to be identified as candidate aminoglycoside-permeant channel (Myrdal and Steyger, 2005). TRPV1 has a pore diameter of ∼1 nm (Chung et al., 2008; Jara-Oseguera et al., 2008) and can be dilated by agonists (Bautista and Julius, 2008; Moiseenkova-Bell et al., 2008). TRPV1 is activated by high temperatures (>43°C), capsaicin, and protons, see **Table 2** (Caterina et al., 1997; Vellani et al., 2001). Cell lines expressing TRPV1 co-incubated with capsaicin and streptomycin undergo rapid cell death (Caterina et al., 1997), suggestive of TRPV1-facilitation of aminoglycoside-induced cytotoxicity. TRPV1 is expressed in the cuticular plate, stereocilia, and cell bodies of hair cells and selected adjacent supporting cells (Zheng et al., 2003), as well as in marginal cells of the stria vascularis (Jiang et al., 2015). Thus, TRPV1 is expressed at key locations along the strial and endolymphatic trafficking route (**Figure 4**).

Involvement of TRPV1 in inflammation is well documented (Davis et al., 2000). Pro-inflammatory mediators up-regulate TRPV1 expression in chronic inflammatory diseases (Engler et al., 2007; Akbar et al., 2008; Cho and Valtschanoff, 2008). Inflammation can also mobilize the translocation of TRPV1 channels from the vesicular reservoir to the plasma membrane via exocytosis (Planells-Cases et al., 2011). Sensitization and translocation of TRPV1 to plasma membrane can also be induced by pro-inflammatory mediators, nerve growth factor and ATP released from damaged cells following tissue trauma (Julius and Basbaum, 2001; Ji et al., 2002; Zhang et al., 2005). Cytokines such as IL-1β, IL-6, and TNFα increase neuronal excitability via TRPV1 (Schafers and Sorkin, 2008; Miller et al., 2009). After kanamycin challenge, TRPV1 expression is unregulated in cochlear and vestibular sensory cells and neuronal ganglia (Kitahara et al., 2005; Ishibashi et al., 2009), and both native and fluorescently tagged gentamicin can permeate TRPV1 (Jiang et al., 2015).

These data suggest that acoustic overstimulation, or systemic inflammation, that induces cochlear expression of cytokines and chemokines, could sensitize or enhance the expression of TRPV1 at key locations to facilitate trafficking of systemically administered aminoglycosides across the stria vascularis into endolymph, as well as into hair cells independently of the MET channel (Li and Steyger, 2011; Li et al., 2011, 2015). Notably, TRPV1 plays a major role in cellular inflammation during cisplatin-induced ototoxicity, as described elsewhere in this Research Topic (Sheth et al., 2017). Whether an intracellular inflammatory-TRPV1 signaling pathway in hair cells occurs during systemic inflammation and/or aminoglycoside cytotoxicity remains to be determined.

### TRPV4

TRPV4 is temperature-sensitive (25–34°C), and mechanically activated by osmotic swelling of cells, as well as by chemically agonists (see **Table 2**), like 4α-phorbol 12,13-didecanoate (Liedtke et al., 2000; Strotmann et al., 2000; Vriens et al., 2004). TRPV4 is expressed by hair cells in the region of the cuticular plate, stereocilia, and cell bodies of hair cells, as well as marginal cells and intermediate cells in the stria vascularis; in addition, TRPV4 is permeable to fluorescently tagged gentamicin (Karasawa et al., 2008). Thus, TRPV4 is expressed at key locations along the strial trafficking route into endolymph and hair cells (**Figure 4**). After kanamycin challenge, the expression of TRPV4 is downregulated in the inner ear sensory cells, neuronal ganglia and stria vascularis (Kitahara et al., 2005; Ishibashi et al., 2009), suggesting that TRPV4 does not enhance cochlear uptake of aminoglycosides during insult, and may represent an otoprotective response. Thus, sepsis-enhanced cochlear uptake of aminoglycosides must overcome any decreased trafficking resulting from inflammatory down-regulated expression of individual aminoglycoside-permeant ion channels.

### TRPC Channels

TRPC3 and TRPC6 are canonical TRP channels expressed by hair cells, with a large (∼6 nm diameter) inner chamber (Mio et al., 2007; Goel and Schilling, 2010; Quick et al., 2012). Endothelial cells also express TRPC6, and activation by phosphoinositides or products downstream of reactive oxygen species induce translocation from the vesicular reservoir to the plasma membrane via exocytosis (Chaudhuri et al., 2016). This results in endothelial inflammation, increased cellular permeability and disrupted barrier function (Tauseef et al., 2012). Similar translocation and activation has been reported for other members of TRP channels too. For example, TRPC4 phosphorylation by Src family tyrosine kinases (STKs) following epidermal growth factor receptor stimulation, induces exocytotic insertion of TRPC4 into the plasma membrane (Odell et al., 2005) and TRPV4 translocation happens after shear stress in primary vascular endothelial cells (Baratchi et al., 2016). Thus, the roles of these TRP channels, and their permeability to aminoglycosides and trafficking across the BLB, especially during inflammation remain to be determined.

### Vasodilation

Vasodilation is a primary consequence of inflammation in order to facilitate extravasation of plasma (i.e., increased paracellular flux) into the interstitial space of tissues. However, in the tight junction-coupled blood-brain barrier and BLB, vasodilation occurs without major increases in paracellular flux. When inflammation-induced vasodilation in the BLB was abrogated in TLR4-hyporesponsive mice, aminoglycoside-uptake by the cochlea was also attenuated (Koo et al., 2015). Conversely, vasodilators like serotonin and ginkgo biloba enhance cochlear uptake of aminoglycosides and cochleotoxicity (Didier et al., 1996; Miman et al., 2002; Koo et al., 2011). Although, these vasodilators have other confounding cochlear effects, it is intriguing that downstream products of reactive oxygen species (e.g., 4-HNE, peroxidized lipids) also dilated cerebral arterioles via activation of TRPA1 (Sullivan et al., 2015). Thus, it will be important to untangle which feature of these polymodal events directly contributes to the increased strial endothelial uptake of aminoglycosides (Koo et al., 2015).

## Neonate-Specific Factors

Most neonates have a continuing maturation of the BLB up to 27 weeks gestational age (GA). Responses to sounds by the fetus can be first detected to 500 Hz tones at 19 weeks GA and increases in frequency range with continuing gestation to 100 to 3000 Hz by 27 weeks GA (Hepper and Shahidullah, 1994). Extrapolating from preclinical data, this suggests that the BLB is largely functionally mature in order to facilitate onset of hearing with the exquisite three-dimensional organization of cochlear fluids and endolymphatic potentials (Ehret, 1976; Yamasaki et al., 2000; Song et al., 2006). This physiological maturation is supported by the co-expression of cubulin and megalin in the apical membranes of marginal cells in the stria vascularis and Reissner’s membrane prior to onset of hearing, as for proximal tubule cells during renal morphogenesis (Christensen and Birn, 2002; Tauris et al., 2009). Neonatal murine pups <2 weeks post-natal age, prior to onset of hearing (Ehret, 1976; Yamasaki et al., 2000; Song et al., 2006), could mimic extremely immature neonates (<27 weeks GA). Neonatal murine pups readily take up fluorescent aminoglycosides compared to adult mice (Dai et al., 2006), however, the effects of this uptake prior to, or during, onset of hearing on mature auditory function remain to be determined.

Substantial evidence demonstrates diminished innate immune responses in neonates to bacterial and viral infections (Levy, 2005), and that individual immune cell types have less capacity to synthesize multiple cytokine responses to immunogenic stimuli. However, empiric data is heterogeneous, with baseline levels and varying immunogenic responses dependent on age, geographical location, race, and TLRs studied (Martino et al., 2012; Georgountzou and Papadopoulos, 2017). The maturing innate immune response during infancy and in specific chronic disease states (e.g., cystic fibrosis) will be an area of immense growth prior to understanding differential effects during developmental maturation of organ systems. Neonates in the NICU may also be exposed to one or more co-therapeutics that can potentiate aminoglycoside-induced hearing loss, including vancomycin, loop diuretics (as an anti-seizure medication), and neuromuscular blocking agents (to facilitate intubation for neonates requiring respiratory assistance), and reviewed by Garinis et al. (2017b). Each of these factors, along with aminoglycoside therapy and inflammation, may contribute to a multiple causative origin of hearing loss proposed for neonates in the NICU (Allegaert et al., 2016).

## Summary

In this review, we explored potential mechanisms by which systemic host-mediated inflammatory responses to immunogenic stimuli could exacerbate aminoglycoside trafficking into the cochlea to enhance aminoglycoside-induced cochleotoxicity. Systemic inflammatory signaling cascades induce cochlear expression of cytokines and chemokines that could modulate the rate of endocytosis, and/or, more likely, sensitize/upregulate the expression of selected aminoglycoside-permeant cation channels within the cochlea, particularly TRPV1. The expression of other (candidate) aminoglycoside-permeant cation channels are down-regulated (TRPV4) or remain unknown (e.g., TRPA1, TRPCs) mean that acquisition of further empirical data is needed. The altered expression and physiology of aminoglycoside-permeant channels should modulate the flux of aminoglycosides across the endothelial cells forming the BLB, through the stria vascularis and into endolymph, and thence into hair cells and supporting cells. Once verified, these mechanisms will be potential targets for novel pharmacotherapeutics that reduce the risk of drug-induced cochleotoxicity and acute kidney damage during systemic inflammation without compromising the required bactericidal efficacy of aminoglycosides.

## Author Contributions

MJ, FT, and PS all conducted the literature review, wrote, revised, edited, and approved submission of the manuscript

## Conflict of Interest Statement

The authors declare that the research was conducted in the absence of any commercial or financial relationships that could be construed as a potential conflict of interest.
